# Progression-free survival as a surrogate endpoint for overall survival in patients with relapsed or refractory multiple myeloma

**DOI:** 10.1186/s12885-024-12263-0

**Published:** 2024-04-29

**Authors:** Meletios Dimopoulos, Pieter Sonneveld, Salomon Manier, Annette Lam, Tito Roccia, Jordan M. Schecter, Patricia Cost, Lida Pacaud, Abbey Poirier, Gabriel Tremblay, Tommy Lan, Satish Valluri, Shaji Kumar

**Affiliations:** 1 Department of Clinical Therapeutics, School of Medicine, National and Kapodistrian University of Athens, Athens, Greece; 2 Erasmus Medical Center, Rotterdam, the Netherlands; 3 University of Lille, Lille, France; 4 Janssen Global Services, Raritan, NJ USA; 5 Janssen Research & Development, Raritan, NJ USA; 6 Legend Biotech USA Inc, Somerset, NJ USA; 7 Cytel Inc. Health Economics & Outcomes Research, Waltham, MA USA; 8 Department of Hematology, Mayo Clinic, Rochester, MN USA

**Keywords:** relapsed/refractory multiple myeloma, Surrogate endpoint, Progression-free survival, Overall survival, Instrumental variable

## Abstract

**Objectives:**

The goal of the research was to assess the quantitative relationship between median progression-free survival (PFS) and median overall survival (OS) specifically among patients with relapsed/refractory multiple myeloma (RRMM) based on published randomized controlled trials (RCTs).

**Methods:**

Two bibliographic databases (PubMed and Embase, 1970–2017) were systematically searched for RCTs in RRMM that reported OS and PFS, followed by an updated search of studies published between 2010 and 2022 in 3 databases (Embase, MEDLINE, and EBM Reviews, 2010–2022). The association between median PFS and median OS was assessed using the nonparametric Spearman rank and parametric Pearson correlation coefficients. Subsequently, the quantitative relationship between PFS and OS was assessed using weighted least-squares regression adjusted for covariates including age, sex, and publication year. Study arms were weighted by the number of patients in each arm.

**Results:**

A total of 31 RCTs (56 treatment arms, 10,450 patients with RRMM) were included in the analysis. The average median PFS and median OS were 7.1 months (SD 5.5) and 28.1 months (SD 11.8), respectively. The Spearman and Pearson correlation coefficients between median PFS and median OS were 0.80 (*P* < 0.0001) and 0.79 (*P* < 0.0001), respectively. In individual treatment arms of RRMM trials, each 1-month increase in median PFS was associated with a 1.72-month (95% CI 1.26–2.17) increase in median OS.

**Conclusion:**

Analysis of the relationship between PFS and OS incorporating more recent studies in RRMM further substantiates the use of PFS to predict OS in RRMM.

**Supplementary Information:**

The online version contains supplementary material available at 10.1186/s12885-024-12263-0.

## Introduction

Multiple myeloma (MM) remains largely incurable, with research focused on finding more effective treatments that can delay disease progression and extend survival [1]. Much progress has been made in the past 20 years. Real-world studies showed that the 5-year survival rate increased from 37 to 62% (years 2000–2019) in a German population [2] and increased from 27 to 47% (years 1994–2016) in a Spanish population [3]. These successes can be attributed to the development of more effective therapies; however, patients will eventually develop relapsed and/or refractory MM (RRMM) and require further therapy [4, 5].

Overall survival (OS) is the gold standard and the most clinically meaningful endpoint in cancer trials from the perspective of health technology assessment (HTA) agencies and payers because it is an objective and straightforward measure of survival benefits provided by a treatment [6, 7]. However, due to necessary extensive follow-up and the confounding effects of subsequent therapy, demonstrating real OS benefits in a timely manner is challenging [6, 8–11]. In recent clinical trials, median OS was not reached with several newer treatments even after a median follow-up of 2–4 years in RRMM and > 6 years in newly diagnosed MM [12–15]. Thus, the improved efficacy of novel therapies is diminishing the feasibility of using OS as an endpoint from the perspective of timeliness and cost. Shifting the focus to shorter term outcomes as predictors of longer term benefit is essential to support early access to new and effective treatments [6]. Progression-free survival (PFS) can be a useful indicator of clinical benefit as it is available earlier than OS and is not influenced by subsequent treatments [16–19]. Indeed, PFS is accepted in multiple institutions, including the United States Food and Drug Administration, European Medicines Agency, and some reimbursement bodies (e.g., National Institute for Health and Care Excellence), as a measure of efficacy in hematological oncology trials [20, 21].

Studies quantitatively analyzing the relationship between PFS and OS among patients with MM are limited, but a positive correlation has been reported between observed treatment effects on PFS with OS [22]. Félix et al. [23] directly estimated the quantitative relationship between median time-dependent endpoints (PFS, event-free survival, and time to progression) and median OS in heterogenous populations with MM. In their analysis of 153 studies with 230 treatment arms published from 1970 to 2011, they found that the correlation coefficient of median PFS with median OS observed in individual treatment arms was 0.75 (*P* < 0.0001); for each additional time-dependent endpoint, a 1.82-month (95% CI 1.6–2.1) increase in median OS was estimated [23]. These estimated coefficients have been recently used in a cost-effectiveness model developed by the Institute for Clinical and Economic Review (a United States-based HTA) to overcome the limitation of having immature OS data [24, 25].

While the analyses by Félix et al. [23] and Dimopoulos et al. [26] used an instrumental variable approach, we reassessed the quantitative relationship between median PFS and median OS by applying a weighted least-squares (WLS) regression [27], an analysis method that does not rely on reporting of the 12-month OS rate. We also restricted our analysis to randomized controlled trials (RCTs) of patients with relapsed or refractory disease.

## Methods

### Search strategy and selection criteria

Two bibliographic databases (PubMed and Embase) were systematically searched for studies published from 1970 to 2017, followed by an updated search of the Embase, MEDLINE, and Evidence-Based Medicine Reviews databases available through the Ovid platform for studies published between 2010 and 2022 (coverage includes ACP Journal Club–American College of Physicians, Database of Abstracts of Reviews of Effects, Cochrane Clinical Answers, Cochrane Central Register of Controlled Trials, Cochrane Methodology Register, Health Technology Assessments, National Health Service Economic Evaluation Database). The search syntaxes are provided in Supplementary Table 1. The analysis was restricted to RCTs conducted in patients with RRMM that reported data on median PFS and median OS [23]. Additionally, data on authors, publication year, primary endpoints, country, intervention used, number of patients, percentage of male patients, median age, and study results were extracted.

## Statistical methods

Each study arm represented 1 observation. The association between median PFS and median OS was assessed using the nonparametric Spearman rank coefficient and the parametric Pearson correlation coefficient, which are widely chosen tests measuring the strength and direction of the association between 2 ranked variables [27]. The definition of PFS may differ between trials, especially regarding the inclusion and exclusion of death as an event, and there can be unobservable trial-related characteristics confounding the relationship between PFS and OS [21]. These may result in estimation bias since the main regressor of interest (PFS) is endogenous [28]. In Félix et al. [23] the instrumental variable approach with 2-stage least-squares regression was originally proposed as an alternative. However, this approach requires the reporting of 12-month OS rates, which several studies did not report [29]. Because the sample size was larger in the analysis by Félix et al. [23] due to inclusion of other types of studies (e.g., non-RRMM and non-RCT), excluding studies not reporting OS data had less impact, whereas our more focused analysis had a smaller sample size that was expected to be impacted by these exclusions. Furthermore, using the 12-month OS rate as an instrumental variable was problematic as it does not meet all 3 criteria of an instrumental variable: (1) relevance assumption: instrument (12-month OS) is causally associated with PFS—not met; (2) exclusion restriction: instrument affects longer term OS only through PFS—not met; (3) exchangeability assumption: instrument is not associated with any confounders (known or unknown) of the association between PFS and longer term OS—met [30]. Therefore, we instead assessed the quantitative relationship between PFS and OS using WLS regression analysis, as it does not rely on reporting of the 12-month OS rate [31]. The restriction of our analysis to RCTs also reduces the variability in definitions of PFS across trials, compared with a dataset of mixed trial designs.

In our analysis, the median OS was regressed on the median PFS controlling for publication year, median age, and sex distribution of the patients, and study arms were weighted by the number of patients in each arm. Additionally, an unadjusted model was conducted with only PFS as the independent variable. The standard error of the resulting coefficient was estimated by the White estimator (robust to heteroscedasticity). This approach was justified by several relevant statistical tests. All the analyses were performed with R version 4.2.2 within the RStudio environment.

## Results

### Sample characteristics

A total of 35 RCTs published between 2006 and 2022 in patients with RRMM were retrieved, of which 31—containing 56 treatment arms and 10,450 patients—were included in the analysis (Supplementary Fig. 1). Each treatment arm included 22–465 patients and the average male proportion was 56.0%, with an average median age of 65 years (Table 1). The average median OS across the unadjusted pooled dataset was 28.1 months (range 8.1–53.6), and the average median PFS was 7.1 months (range 1.7–26.1). Additional details about the 31 studies included in the analysis are provided in Supplementary Table 2.

**Table 1:** Characteristics of the 56 arms^a^ (31 studies) included in the analysis

Variable	Mean	Weighted Mean^b^	Median	Min	Max	SD
Sample size^c^	186.6	–	146.0	22.0	465.0	128.4
Publication year	2016.2	2016.6	2017.5	2006.0	2022.0	5.0
Median age^d^, year	65.0	64.7	65.0	59.0	71.0	2.7
Male, %	56.0	55.8	56.8	36.8	70.0	6.1
OS, months	27.8	32.1	28.1	8.1	53.6	11.8
PFS, months	8.6	10.3	7.1	1.7	26.1	5.5

^a^Analysis only included the study arms in which both median PFS and median OS were observed

^b^Weighted mean is calculated using sample size of each arm as weight

^c^Sample size refers to the number of patients included in each treatment arm

^d^Most of the studies reported the median age instead of the mean age of the study population

Min, minimum value; Max, maximum value; OS, overall survival; PFS, progression-free survival; SD, standard deviation

### Correlation between PFS and OS

**Fig. 1:**
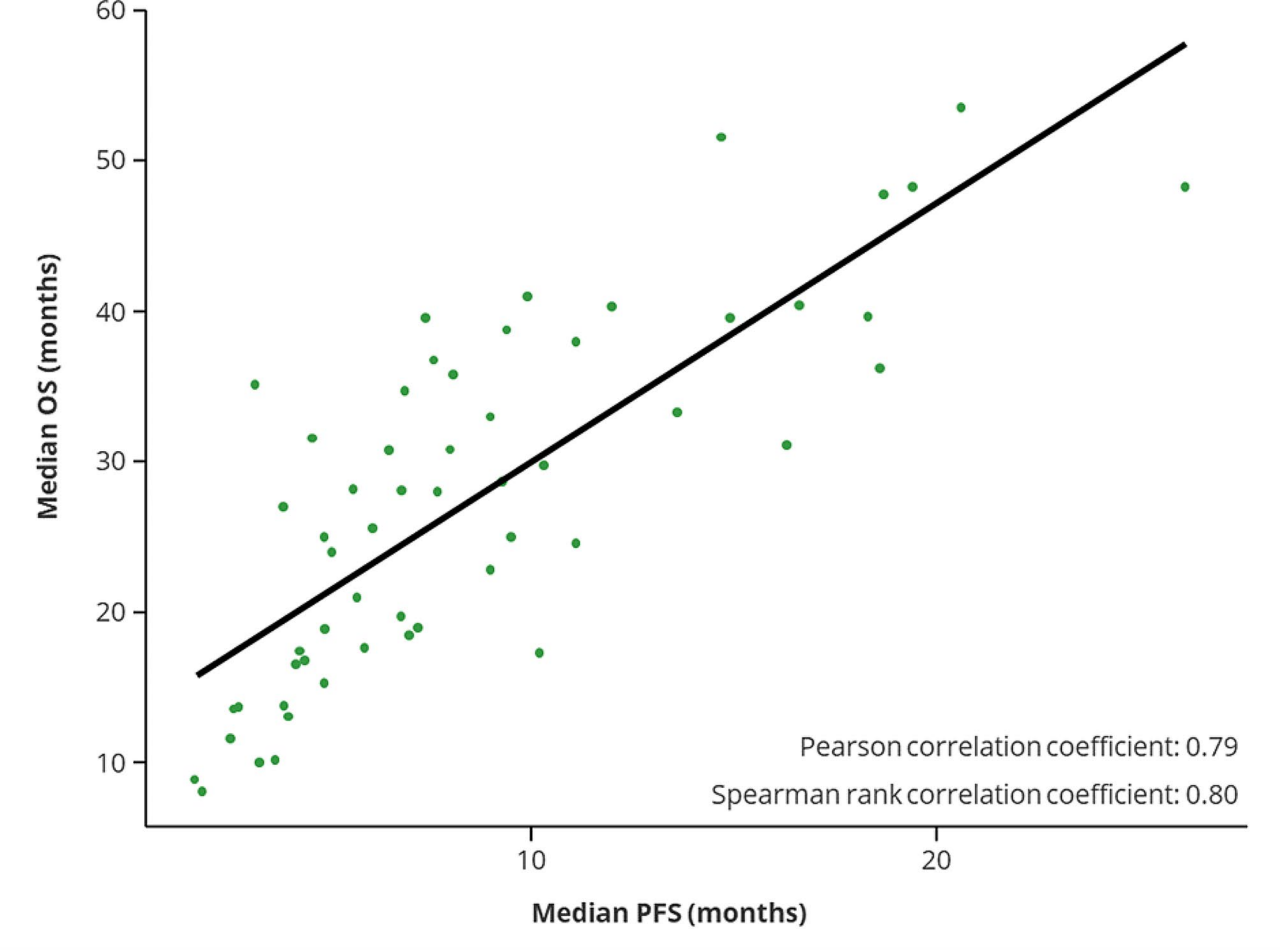
Association between median PFS and median OS in individual treatment arms. Each dot represents a study arm. OS, overall survival; PFS, progression-free survival

The estimated Spearman rank and Pearson correlation coefficients between median PFS and median OS in RRMM were 0.80 (*P* < 0.0001) and 0.79 (*P* < 0.0001), respectively, suggesting a strong correlation. The positive correlation between median PFS and median OS was relatively consistent across all available study arms. However, the relationship was not perfectly linear, especially when the OS and PFS values were small (Fig. 1).

### Modeling the quantitative relationship between PFS and OS

The effects of median PFS on median OS estimated from the WLS are reported in Table 2. Each 1-month increase in median PFS was associated with a 1.72-month (95% CI 1.26–2.17) increase in median OS. All other covariates in the regression (age, proportion of male patients, and publication year) were not statistically significant, suggesting that the PFS was the main explanatory factor in this relationship. A sensitivity analysis confirmed that when no covariates were included in the model, the effect estimate of PFS was similar (1.69; 95% CI 1.27–2.12) (Table 3). Inclusion of study arms for which age and sex of study patients were unknown also did not appreciably affect the impact of PFS on OS.

**Table 2:** Effect of median PFS on median OS estimated from the weighted least-squares regression

Covariables	Coefficients	*P* value	95% confidence interval
PFS, months	1.72	< 0.001	1.26, 2.17
Median age, years	–0.38	0.42	–1.33, 0.56
Male, %	–5.07	0.80	–45.29, 35.15
Publication year	–0.08	0.75	–0.57, 0.41
Constant	200.11	0.67	–748.58, 1148.80

OS, overall survival; PFS, progression-free survival

**Table 3:** Sensitivity analysis

Model	Median OS ~ Median PFS (Model including covariates median age, male proportion, and publication year)		Median OS ~ Median PFS^a^ (Model not including covariates)	
Variable	Main analysis	Arms with unknown age and sex imputed^b^	Same input data as main analysis	Input data include arms with unknown age and sex
Median PFS (95% CI)	1.72 (1.26, 2.17)	1.70 (1.27, 2.14)	1.69 (1.27, 2.12)	1.71 (1.29, 2.13)
Median age (95% CI)	–0.38 (–1.33, 0.56)	–0.52 (–1.38, 0.34)	–	–
Male, % (95% CI)	–5.07 (–45.29, 35.15)	3.13 (–31.27, 37.53)	–	–
Publication year (95% CI)	–0.08 (–0.57, 0.41)	0.08 (–0.35, 0.51)	–	–
Constant (Intercept)	200.11 (–748.58, 1148.80)	–107.66 (–940.93, 725.62)	14.73 (10.24, 19.23)	14.33 (10.01, 18.64)

^a^Median PFS is the only independent variable in this model, no other covariates were included

^b^Median age and male proportion was imputed using weighted mean

OS, overall survival; PFS, progression-free survival

## Discussion

The choice of optimal endpoints for clinical trials is becoming increasingly important with pressure from HTAs and payers to show that treatment leads to tangible clinical benefits [32–34]. Using PFS as a surrogate endpoint in clinical trials rather than waiting for OS data to become available can accelerate the availability of drugs for patients through the earlier reporting of results and facilitate rapid introduction of new therapies into clinical practice [35, 36]. We addressed a gap in evidence for using PFS as a surrogate endpoint for OS, specifically in patients with RRMM treated with modern therapies. Our analysis validated PFS as a surrogate endpoint for OS in this population and showed that each 1-month increase in median PFS was associated with a 1.26- to 2.17-month (median 1.72) increase in median OS, when the covariates median age, male proportion, and publication year were kept constant. The regression coefficient of 1.72 estimated in the WLS regression model is similar to that reported by Félix et al. [23] (1.82) for the base model adjusted for age, sex, and year of publication. The correlation coefficient in our analysis (0.80) is consistent with the positive relationship between PFS and OS reported previously [22].

There are several key differences between our analysis and the one by Félix et al. [23]: (1) ours included RCTs only (vs. pooling RCTs and uncontrolled studies), resulting in a relatively more consistent definition of PFS across studies; (2) ours included patients with RRMM only (vs. all MM patients); (3) ours used the WLS method (vs. the instrumental variable method requiring 12-month OS rates); and (4) ours included PFS only (vs. multiple time-dependent endpoints) and thus provides a more stringent quantitative correlation between median PFS and median OS.

Our results are valuable for indirect treatment comparisons and economic assessment of new MM therapies. In order to use PFS as a surrogate for OS to evaluate an experimental treatment in clinical trials, a formal validation should occur to show it can fully capture the net effect of treatment on OS [6, 37]. A future step will be to evaluate the association between the treatment effect with both, the PFS and the OS. Treatment effect can be either absolute difference in median survival times or hazard ratios.

While the study demonstrates several strengths, there are a few limitations that need to be considered. The results apply to RRMM only and are not reflective of the relationship between PFS and OS for MM overall: the PFS-OS relationship in patients with newly diagnosed MM may be different than in patients with RRMM since newly diagnosed MM is slower to progress and has different disease dynamics than RRMM [38–40]. Studies that had median PFS data but did not reach median OS were excluded from the analysis, as were studies in which required data were distributed across multiple publications. The analysis included studies with differing definitions of PFS. Also, published studies were selected from 2 systematic literature reviews with different selection criteria, and study arms with missing information on covariates, median PFS, or median OS were excluded. Although RCTs have the most rigid design and reliable data collection, other study designs may need further investigation considering the expanding use of real-world studies in evidence generation. Finally, this analysis was based on aggregated data obtained from existing studies; however, more data could be obtained by collecting individual-level data, thereby providing more statistical validity [41].

This analysis only included study arms where both median PFS and median OS were observed (i.e., 56 study arms). Due to short follow-up periods, several arms in which median OS was not observed were excluded (i.e., censoring) from the analysis. The feasibility of using only uncensored observations relies on the assumption that the data were missing at random [42]. Félix et al. [23] found in a sensitivity analysis of their data that the estimated adjusted effect of median PFS on median OS when only uncensored observations were included (coefficient = 2.62, 95% CI 1.52–3.71) was close to the adjusted effect when all observations were included (coefficient = 2.45, 95% CI 1.71–3.20). This suggests that in the RRMM analysis, results with only uncensored observations are close to those using censored observations. In the Félix et al. study, differences between the regression coefficients may be due to the difference in the proportion of RCTs between uncensored and total observations [23]. Since only RCTs were included in our analysis, the difference caused by study design was unlikely, and an assumption was made that the data were missing at random. Furthermore, our sensitivity analysis included study arms that were missing data on patient age and sex which showed minimal impact on the relationship between PFS and OS.

This analysis included all the treatments for RRMM in which eligible RCTs were available until 2022. These results should be interpreted as the average association between PFS and OS among all retrieved trials after controlling for year and trial characteristics and therefore may not be directly observed in any single new-agent trial.

## Conclusions

Studies published from 2006 to 2022 in RRMM further substantiate evidence supporting the use of PFS to predict OS in RRMM.

### Electronic supplementary material

Below is the link to the electronic supplementary material.

Supplementary Material 1

Supplementary Material 2

Supplementary Material 3

## Data Availability

The key data generated or analyzed during this study are included in this published article (supplementary material). The full dataset used and/or analyzed during this study are available from the corresponding author on reasonable request.

## Abbreviations

PFS: Progression-free survival
OS: Overall survival
MM: Multiple myeloma
RRMM: Relapsed/refractory multiple myeloma
RCT: Randomized controlled trial
HTA: Health technology assessment
WLS: Weighted least squares
