# Supplementary material for: Progression-free survival as a surrogate endpoint for overall survival in patients with relapsed or refractory multiple myeloma
Source: BMC Cancer. 2024 Apr 29;24:541. doi: 10.1186/s12885-024-12263-0 (PMC11057089; doi:10.1186/s12885-024-12263-0)
Supplement: Supplementary file 1 — Supplementary Material 1 [file 12885_2024_12263_MOESM1_ESM.docx]

**Additional Table 2. List of included studies and extracted data**

| **Author** | **Year** | **Arm** | **Interventions** | **Sample size** | **Median age (years)** | **Male proportion** | **Median PFS (months)** | **Median OS (months)** |
| --- | --- | --- | --- | --- | --- | --- | --- | --- |
| Friedenberg | 2006 | 1 | VAD | 48 | 61 | 0.62 | 7 | 18.5 |
|  | 2006 | 2 | VAD + Val | 46 | 67 | 0.7 | 4.9 | 15.3 |
| Richardson | 2006 | 1 | Len (15 mg) | 35 | 59 | 0.6 | 3.9 | 27 |
|  | 2006 | 2 | Len (30 mg) | 67 | 60 | 0.64 | 7.7 | 28 |
| Orlowski | 2007 | 1 | Bor + PLD | 324 | 61 | 0.58 | 9 | 33 |
|  | 2007 | 2 | Bor | 322 | 62 | 0.54 | 6.5 | 30.8 |
| Dimopoulos | 2009 | 1 | Dex + Len | 353 | 63 | 0.6 | 11.1 | 38 |
|  | 2009 | 2 | Dex + Placebo | 351 | 63 | 0.59 | 4.6 | 31.6 |
| Arnulf | 2012 | 1 | Bor SC | 148 | 64.5 | 0.5 | 9.3 | 28.7 |
| Garderet | 2012 | 1 | Bor + Thal + Dex | 135 | 60 | 0.64 | 18.3 | 39.6 |
|  | 2012 | 2 | Thal + Dex | 134 | 62.6 | 0.62 | 13.6 | 33.3 |
| Hjorth | 2012 | 1 | Thal + Dex | 67 | 71 | 0.42 | 9 | 22.8 |
|  | 2012 | 2 | Bor + Dex | 64 | 71 | 0.64 | 7.2 | 19 |
| Dimopoulos | 2013 | 1 | Bor | 320 | 63 | 0.58 | 6.8 | 28.1 |
| San-Miguel & Dimopoulos | 2013 | 1 | Pom + Dex  (low dose) | 302 | 64 | 0.6 | 4 | 13.1 |
|  | 2013 | 2 | Dex (high dose) | 153 | 65 | 0.57 | 1.9 | 8.1 |
| White | 2013 | 1 | Bor + Placebo | 53 | 65 | 0.57 | 5.1 | 24 |
| Richardson | 2014 | 1 | Pom + Dex  (low dose) | 113 | 64 | 0.55 | 4.2 | 16.5 |
|  | 2014 | 2 | Pom | 108 | 61 | 0.53 | 2.7 | 13.6 |
| Orlowski | 2015 | 1 | Sil + Bor | 142 | 64 | 0.51 | 8 | 30.8 |
|  | 2015 | 2 | Placebo + Bor | 144 | 61 | 0.59 | 7.6 | 36.8 |
| Baz | 2016 | 1 | Pom + Dex | 36 | 64 | 0.64 | 4.4 | 16.8 |
| Jakubowiak | 2016 | 1 | Placebo + Bor + Dex | 75 | 65 | 0.49 | 6.9 | 34.7 |
| San-Miguel | 2016 | 1 | Pano + Bor + Dex | 387 | 63 | 0.52 | 11.99 | 40.3 |
|  | 2016 | 2 | Placebo + Bor + Dex | 381 | 63 | 0.54 | 8.08 | 35.8 |
| Hájek | 2017 | 1 | Car | 157 | 63 | 0.52 | 3.7 | 10.2 |
|  | 2017 | 2 | COR + CYC (optional) | 158 | 66 | 0.61 | 3.3 | 10 |
| Kropff | 2017 | 1 | Bor + Dex + CYC | 47 | 70.7 | 0.55 | 9.9 | 41 |
| Lida | 2018 | 1 | Thal + Dex | 22 | 66.5 | 0.59 | 3.2 | 35.2 |
| Stewart & Siegel | 2018 | 1 | Car + Len + Dex | 396 | 64 | 0.54 | 26.1 | 48.3 |
|  | 2018 | 2 | Len + Dex | 396 | 65 | 0.59 | 16.6 | 40.4 |
| Dimopoulos & Orlowski | 2019 | 1 | Car + Dex | 464 | 65 | 0.52 | 18.7 | 47.8 |
|  | 2019 | 2 | Bor + Dex | 465 | 65 | 0.49 | 9.4 | 38.8 |
| Mateos | 2019 | 1 | Pem + Pom + Dex | 126 | 65 | 0.62 | 5.7 | 21 |
|  | 2019 | 2 | Pom + Dex | 125 | 67 | 0.63 | 7.4 | 39.6 |
| Spicka | 2019 | 1 | PLI + Dex | 171 | 64 | 0.57 | 2.6 | 11.6 |
|  | 2019 | 2 | Dex | 84 | 65 | 0.42 | 1.7 | 8.9 |
| Grosicki | 2020 | 1 | Bor + Dex | 207 | 67 | 0.56 | 9.5 | 25 |
| Lonial & Dimopoulos | 2020 | 1 | Elo + Len + Dex | 321 | 67 | 0.60 | 19.4 | 48.3 |
|  | 2020 | 2 | Len + Dex | 325 | 66 | 0.59 | 14.9 | 39.6 |
| Lonial | 2020 | 1 | BELMA (2.5 mg/kg) | 97 | 65 | 0.53 | 2.8 | 13.7 |
|  | 2020 | 2 | BELMA (3.4 mg/kg) | 99 | 67 | 0.57 | 3.9 | 13.8 |
| Montefusco | 2020 | 1 | Bor + CYC + Dex | 76 | 65 | 0.37 | 16.3 | 31.1 |
|  | 2020 | 2 | Len + CYC + Dex | 79 | 63.6 | 0.56 | 18.6 | 36.2 |
| Dimopoulos | 2021 | 1 | Elo + Pom + Dex | 60 | 69 | 0.53 | 10.3 | 29.8 |
|  | 2021 | 2 | Pom + Dex | 57 | 66 | 0.61 | 4.3 | 17.4 |
|  | 2021 | 3 | Isa + Dex | 55 | 66 | 0.53 | 10.2 | 17.3 |
|  | 2021 | 4 | Isa | 109 | 68 | 0.47 | 4.9 | 18.9 |
| Moreau & Richardson OK this | 2021 | 1 | Ixa + Len + Dex | 360 | 66 | 0.58 | 20.6 | 53.6 |
|  | 2021 | 2 | Placebo + Len + Dex | 362 | 66 | 0.56 | 14.7 | 51.6 |
| Richardson | 2022 | 1 | Isa + Pom + Dex | 154 | 68 | 0.58 | 11.1 | 24.6 |
|  | 2022 | 2 | Pom + Dex | 153 | 66 | 0.46 | 5.9 | 17.7 |
| Schjesvold | 2022 | 1 | Melflufen + Dex | 246 | 68 | 0.57 | 6.8 | 19.8 |
|  | 2022 | 2 | Pom + Dex | 249 | 68 | 0.56 | 4.9 | 25 |
| Usmani | 2022 | 1 | Daratumumab SC | 263 | 65 | 0.52 | 5.6 | 28.2 |
|  | 2022 | 2 | Daratumumab IV | 259 | 68 | 0.58 | 6.1 | 25.6 |

BELMA, belantamab mafodotin; Bor, bortezomib; Car, carfilzomib; COR, corticosteroids; CYC, cyclophosphamide; Dex, dexamethasone; Elo, elotuzumab; Isa, isatuximab; IV, intravenous; Ixa, ixazomib; Len, lenalidomide; OS, overall survival; Pano, panobinostat; Pem, pembrolizumab; PFS, progression-free survival; PLD, pegylated liposomal doxorubicin; PLI, plitidepsin; Pom, pomalidomide; SC, subcutaneous; Sil, siltuximab; Thal, thalidomide; VAD, vincristine, doxorubicin, and dexamethasone; Val, valspordar.
