# Supplementary material for: Progression-free survival as a surrogate endpoint for overall survival in patients with relapsed or refractory multiple myeloma
Source: BMC Cancer. 2024 Apr 29;24:541. doi: 10.1186/s12885-024-12263-0 (PMC11057089; doi:10.1186/s12885-024-12263-0)
Supplement: Supplementary file 2 — Supplementary Material 2 [file 12885_2024_12263_MOESM2_ESM.docx]

**Additional Table 1. Searching syntax**

| **Search conducted** | | **October 30, 2022** | |
| --- | --- | --- | --- |
| **Databases searched** | | EBM Reviews – Cochrane Database of Systematic Reviews | 2005 to October 26, 2022 |
|  |  | EBM Reviews – ACP Journal Club | 1991 to October 2022 |
|  |  | EBM Reviews – Database of Abstracts of Reviews of Effects | 1st Quarter 2016 |
|  |  | EBM Reviews – Cochrane Clinical Answers | October 2022 |
|  |  | EBM Reviews – Cochrane Central Register of Controlled Trials | September 2022 |
|  |  | EBM Reviews – Cochrane Methodology Register | 3rd Quarter 2012 |
|  |  | EBM Reviews – Health Technology Assessment | 4th Quarter 2016 |
|  |  | EBM Reviews – NHS Economic Evaluation Database | 1st Quarter 2016 |
|  |  | Embase | 1974 to October 28, 2022 |
|  |  | Ovid MEDLINE(R) ALL | 1946 to October 27, 2022 |
| **Term** | | | |
| 1 | multiple myeloma/ | | |
| 2 | myeloma/ or multiple myeloma/ | | |
| 3 | (myeloma$ or multiple-myeloma$ or Kahler$).ti,ab. | | |
| 4 | or/1-3 | | |
| 5 | exp Recurrence/ | | |
| 6 | exp cancer recurrence/ or exp relapse/ or exp recurrent disease/ | | |
| 7 | (relap$ OR refract$ OR resist$ OR persist$ OR return$ OR reoccur$ OR reocur$ OR (re adj2 occur) OR (re adj2 ocur$) OR recurren$ OR salvage$ or RRMM).mp,af,tw. | | |
| 8 | (prior or progress$ or (previously adj3 treat$) or (previously adj2 receiv$) or pretreat$ or fail$ or unrespon$).mp,af,tw. | | |
| 9 | or/5-8 | | |
| 10 | 4 and 9 | | |
| 11 | Bortezomib/ or Lenalidomide/ or Panobinostat/ or Vorinostat/ or Bendamustine Hydrochloride/ or Nivolumab/ or Receptors, Chimeric Antigen/ or Melphalan/ or Leflunomide/ or Azacitidine/ or Dasatinib/ or Crizotinib/ or Afatinib/ or Ado-Trastuzumab Emtansine/ or Ipilimumab/ or B-Cell Maturation Antigen/ or Antibodies, Bispecific/ or Mitoxantrone/ | | |
| 12 | bortezomib/ or lenalidomide/ or ixazomib/ or daratumumab/ or carfilzomib/ or pomalidomide/ or panobinostat/ or elotuzumab/ or selinexor/ or melphalan flufenamide/ or melphalan/ | | |
| 13 | vorinostat/ or isatuximab/ or bendamustine/ or belantamab/ or belantamab mafodotin/ or encorafenib/ or binimetinib/ or pembrolizumab/ or nivolumab/ or erdafitinib/ or idecabtagene vicleucel/ or ciltacabtagene autoleucel/ or chimeric antigen receptor/ or iberdomide/ or elranatamab/ or teclistamab/ or magrolimab/ or leflunomide/ or azacitidine/ or talquetamab/ or trametinib/ or dasatinib/ or dabrafenib/ or defactinib/ or binimetinib/ or crizotinib/ or taselisib/ or afatinib/ or capivasertib/ or trastuzumab emtansine/ or ipilimumab/ or nirogacestat/ or mezagitamab/ or B cell maturation antigen/ or bispecific antibody/ or zimberelimab/ or duvelisib/ or venetoclax/ or pirtobrutinib/ or lemzoparlimab/ or eftozanermin alfa/ or cevostamab/ or ciforadenant/ or tasquinimod/ or mitoxantrone/ or lisaftoclax/ or subasumstat/ or relatlimab/ or belantamab/ or belantamab mafodotin/ or tocilizumab/ or catequentinib/ or tazemetostat/ or modakafusp alfa/ or pelareorep/ | | |
| 14 | (bortezomib or velcade$ or lenalidomide or revlimid$ or revimid$ or ixazomib or ninlaro$ or daratumumab or darzalex$ or carfilzomib or kyprolis$ or pomalidomide or pomalyst$ or imnovid$ or panobinostat or farydak$ or elotuzumab or empliciti$ or selinexor or xpovio$).ti,ab | | |
| 15 | (melflufen or melphalan or Merphalan or J1 or J-1 or "J 1" or alkeran$ or evomela$ or PEPAXTO$ or Vorinostat or Zolinza$ or isatuximab$ or Sarclisa$ or bendamustin$ or Bendeka$ or Treanda$ or Belrapzo$ or Ribomustin$ or Cytostasan or TJ202$ or MOR03087 or MOR202$ or Belantamab or BLENREP$ or GSK2857916 or GSK 2857916 or GSK-2857916 or Encorafenib or Braftovi$ or Binimetinib or Mektovi$ or Pembrolizumab or lambrolizumab or Keytruda$ or nivolumab or OPDIVO$ or erdafitinib or Balversa$ or JNJ-42756493 or JNJ42756493 or JNJ 42756493 or Ciltacabtagene Autoleucel or cilta-cel or ciltacel or cilta cel or CARVYKTI$ or JNJ-68284528 or JNJ68284528 or JNJ 68284528 or LCAR-B38M or LCARB38M or LCAR B38M or RAPA-201 or RAPA201 or RAPA 201 or idecabtagene vicleucel or ide-cel or idecel or ide cel or ABECMA$ or bb2121 or bb 2121 or bb-2121).ti,ab. | | |
| 16 | (Iberdomide or cc220 or cc-220 or Elranatamab or PF-06863135 or PF06863135 or Teclistamab or jnj64007957 or jnj-64007957 or Magrolimab or Leflunomide or ARAVA$ or Azacitidine or VIDAZA$ or CC-486 or CC486 or Talquetamab or Trametinib or MEKINIST$ or GSK1120212 or GSK-1120212 or JTP74057 or JTP-74057 or Dasatinib or SPRYCEL$ or BMS-354825 or BMS354825 or Dabrafenib or TAFINLAR$ or GSK2118436 or GSK-2118436 or Defactinib or VS-6063 or VS6063 or Binimetinib or MEKTOVI$ or MEK162 or MEK-162 or Crizotinib or XALKORI$ or PF2341066 or PF-2341066 or AZD4547 or AZD-4547 or AZD1775 or AZD-1775 or GSK2636771 or GSK-2636771 or Taselisib or GDC-0032 or GDC0032 or Afatinib or GILOTRIF$ or Capivasertib or AZD5363 or AZD-5363 or "Trastuzumab Emtansine" or Ado-Trastuzumab or AdoTrastuzumab or KADCYLA$ or Lisaftoclax or APG-2575 or APG2575 or AO-176 or AO176 or Ipilimumab or YERVOY$ or BGB-11417 or BGB11417 or Nirogacestat or PF-03084014 or PF03084014 or TAK-981 or TAK981 or Mezagitamab or TAK079 or TAK-079 or GEN3014 or GEN-3014 or HexaBody$ or BMS-986016 or BMS986016 or BMS-986207 or BMS986207 or PHE885 or PHE-885 or Descartes-08 or Descartes08 or Descartes-11 or Descartes11 or Descartes-25 or Descartes25 or CT103A or CT-103A or SLAMF7 or CS-1 or CS1 or OPC-415 or OPC415 or MMG49 or MMG-49 or ALLO-605 or ALLO605 or NEXI-002 or NEXI002 or PBCAR269A or PBCAR-269A or ARI0002h or ARI-0002h or EMB-06 or EMB06 or HDP-101 or HDP101 or HPN217 or HPN-217 or Subasumstat or Relatlimab).ti,ab. | | |
| 17 | (C-CAR088 or CCAR088 or LNA-i-Mir-221 or LNAiMir-221 or LNAiMir221 or AB308 or AB-308 or Zimberelimab or AB122 or AB-122 or COM902 or COM-902 or COM701 or COM-701 or Duvelisib or COPIKTRA$ or IPI-145 or IPI145 or Venetoclax or VENCLEXTA$ or venclyxto$ or Pirtobrutinib or loxo-305 or loxo305 or loxo-338 or loxo338 or GC012F or GC-012F or IM21 or IM-21 or STI-1492 or STI1492 or NMS-03597812 or NMS03597812 or GPRC5D or CC-95266 or CC95266 or CID-103 or CID103 or ION251 or ION-251 or REGN5458 or REGN-5458 or C-4-29 or C429 or CTX120 or CTX-120 or CC-92328 or CC92328 or APRIL$ or Lemzoparlimab or CC-98633 or CC98633 or Eftozanermin or ABBV-621 or ABBV621 or FT576 or FT-576 or CXCR4 or AGENT-797 or AGENT797 or FHND6091 or FHND-6091 or AEVI-007 or AEVI007 or JNJ-64407564 or JNJ64407564 or jnj-7564 or jnj7564 or TTI-621 or TTI621 or TTI-622 or TTI622 or XMAB24306 or XMAB-24306 or Cevostamab or BFCR4350A or BFCR-4350A or MCARH109 or MCARH-109 or AMG701 or AMG-701 or Ciforadenant or CPD-DARA or CPDDARA or RO7425781 or RO-7425781 or OPD5 or INCB053914 or INCB-053914 or RO7297089 or RO-7297089 or TriPRIL$ or ABBV-383 or ABBV383 or ORIC-533 or ORIC533 or Y150 or Y-150 or CYAD-211 or CYAD211 or CT0590 or CT-0590 or MLN9708 or MLN-9708 or F182112 or F-182112 or TAK-573 or TAK573 or Tasquinimod or abr215050 or abr-215050 or TEG002 or TEG-002 or 211At-OKT10-B10 or spCART-269 or spCART269 or Mitoxantrone or ABBV-467 or ABBV467 or TQB3602 or TQB-3602 or IBI346 or IBI-346 or HB10101 or HB-10101 or JWCAR129 or JWCAR-129).ti,ab. | | |
| 18 | (Tocilizumab or ACTEMRA$ or Anlotinib or CATEQUENTINIB or AL-3818 or AL3818 or Tazemetostat or TAZVERIK$ or Modakafusp or Pelareorep or REOLYSIN$ or BB0209 or BB-0209 or Indium or Actinium or CM336 or CM-336 or CT0591CP or CT-0591CP or CC-92480 or CC92480 or BMS-986158 or BMS986158 or EOS884448 or EOS-884448 or EOS-448 or EOS448 or GSK4428859A or GSK-4428859A or HRS-3738 or HRS3738 or ISB-1442 or ISB1442 or ABBV-453 or ABBV453 or STI-6129 or STI6129 or LMY-920 or LMY920 or LCAR-BCDR or LCARBCDR or OriC321 or OriC-321 or CART-38 or CART38 or CART-BCMA or CARTBCMA or CART-ddBMCA or CARTddBMCA or LUCAR-B68 or LUCARB68 or MCARH125 or MCARH-125 or P-BCMA-ALLO1 or PBCMAALLO1 or PD1-BCMA-CART or PD1BCMACART).ti,ab. | | |
| 19 | (CAR-T cell$ or CAR-Tcell$ or CAR-T-cell$ or CAR T cell$ or CAR Tcell$ or CAR T-cell$ or CART cell$ or CART-cell$ or CARTcell$ or CAR-T or CART or "CAR T" or Chimeric antigen receptor$ or Artificial T-Cell Receptor$ or Artificial TCell Receptor$ or Artificial T Cell Receptor$ or Chimeric T-Cell Receptor$ or Chimeric TCell Receptor$ or Chimeric T Cell Receptor$ or Chimeric Immunoreceptor$ or B-cell maturation antigen or BCMA$ or bispecific antibody or bi-specific antibody or BAT or Trispecific or Tri-specific or TriTAC).ti,ab. | | |
| 20 | or/11-19 | | |
| 21 | 10 and 20 | | |
| 22 | exp Randomized Controlled Trial/ or exp Random Allocation/ or exp randomization/ | | |
| 23 | exp Placebos/ | | |
| 24 | exp Double-Blind Method/ or exp Single-Blind Method/ | | |
| 25 | exp clinical trial/ or exp clinical trial, phase ii/ or exp clinical trial, phase iii/ or exp controlled clinical trial/ | | |
| 26 | exp controlled clinical trials as topic/ or exp Randomized Controlled Trials as Topic/ or exp clinical trials as topic/ | | |
| 27 | exp Multicenter Study/ | | |
| 28 | exp Randomized Controlled Trial/ or exp Random Allocation/ or exp randomization/ | | |
| 29 | exp placebo/ | | |
| 30 | exp double blind procedure/ or exp single blind procedure/ or exp crossover procedure/ | | |
| 31 | exp clinical trial/ or exp phase 2 clinical trial/ or exp phase 3 clinical trial/ or exp controlled clinical trial/ | | |
| 32 | exp "controlled clinical trial (topic)"/ or exp "clinical trial (topic)"/ or exp "randomized controlled trial (topic)"/ | | |
| 33 | exp multicenter Study/ | | |
| 34 | randomized controlled trial.pt. | | |
| 35 | controlled clinical trial.pt. | | |
| 36 | random$.ti,ab,kw. | | |
| 37 | blind$.ti,ab,kw. | | |
| 38 | (placebo$ or assign$ or allocat$ or volunteer$).ti,ab,kw. | | |
| 39 | (parallel$ or factorial$ or crossover$ or cross over$).ti,ab,kw. | | |
| 40 | ('phase 3' or 'phase 2' or 'phase III' or 'phase II').af. | | |
| 41 | ((single or double or triple) adj3 (blind$ or mask$ or dummy)).af. | | |
| 42 | ('double-blind' or 'double-blinded').af. | | |
| 43 | (open label or open-label).af. | | |
| 44 | ("single arm" or "single-arm" or "single group" or "single-group").ti,ab. | | |
| 45 | exp Meta-Analysis/ or exp Meta-Analysis as Topic/ or exp "Systematic Review"/ | | |
| 46 | exp meta analysis/ or exp "meta analysis (topic)"/ or exp "systematic review"/ | | |
| 47 | (meta analy$ or meta-analy$ or metanaly$ or metaanaly$).ti,ab. | | |
| 48 | ((systematic$ or evidence$) adj3 (review$ or overview$)).ti,ab. | | |
| 49 | ((multiple treatment$ or indirect or mixed) adj2 comparison$).ti,ab. | | |
| 50 | or/22-49 | | |
| 51 | 21 and 50 | | |
| 52 | (newly diagnos$ or newly-diagnos$ or newlydiagnos$ or NDMM or first line or first-line or firstline). ti,ab. | | |
| 53 | (untreat$ or naive or treatment-naive or treatmentnaive).ti,ab. | | |
| 54 | (maintenance or postconsolidation or post-consolidation or consolidation or induction). ti,ab. | | |
| 55 | (addresses or bibliography or biography or case report or comment or congresses or consensus development conference or duplicate publication or editorial or guideline or interview or lectures or letter or monograph or news or practice guideline or "review literature" or "review of reported cases" or review, academic or review, multicase or review, tutorial or twin study). pt. | | |
| 56 | (animals/ not (humans/ and animals/)) or (animal/ not (human/ and animal/)) | | |
| 57 | case report/ or case reports/ | | |
| 58 | epidemiologic studies/ or epidemiology/ | | |
| 59 | exp case-control studies/ or exp case control study/ | | |
| 60 | exp cross-sectional studies/ or exp cross-sectional study/ | | |
| 61 | retrospective studies/ or retrospective study/ | | |
| 62 | observational study/ | | |
| 63 | (case control or case-control or retrospective or cross sectional or cross-sectional or observational).ti,ab. | | |
| 64 | (registry or claims or survey or chart review or real world or real-world or audit).ti,ab. | | |
| 65 | cost-benefit analysis/ or "cost effectiveness analysis"/ or "cost minimization analysis"/ or "cost benefit analysis"/ or "cost utility analysis"/ | | |
| 66 | (cost$ adj2 (effective$ or utilit$ or benefit$ or minimi$ or consequence$)).ti,ab | | |
| 67 | (CEA or CMA or CBA or CUA).ti,ab. | | |
| 68 | or/52-67 | | |
| 69 | 51 not 68 | | |
| 70 | limit 69 to english language | | |
| 71 | limit 70 to humans | | |
| 72 | limit 71 to yr="2022 -Current" | | |
