# Supplementary figures and images for: Progression-free survival as a surrogate endpoint for overall survival in patients with relapsed or refractory multiple myeloma
Source: BMC Cancer. 2024 Apr 29;24:541. doi: 10.1186/s12885-024-12263-0 (PMC11057089; doi:10.1186/s12885-024-12263-0)

**
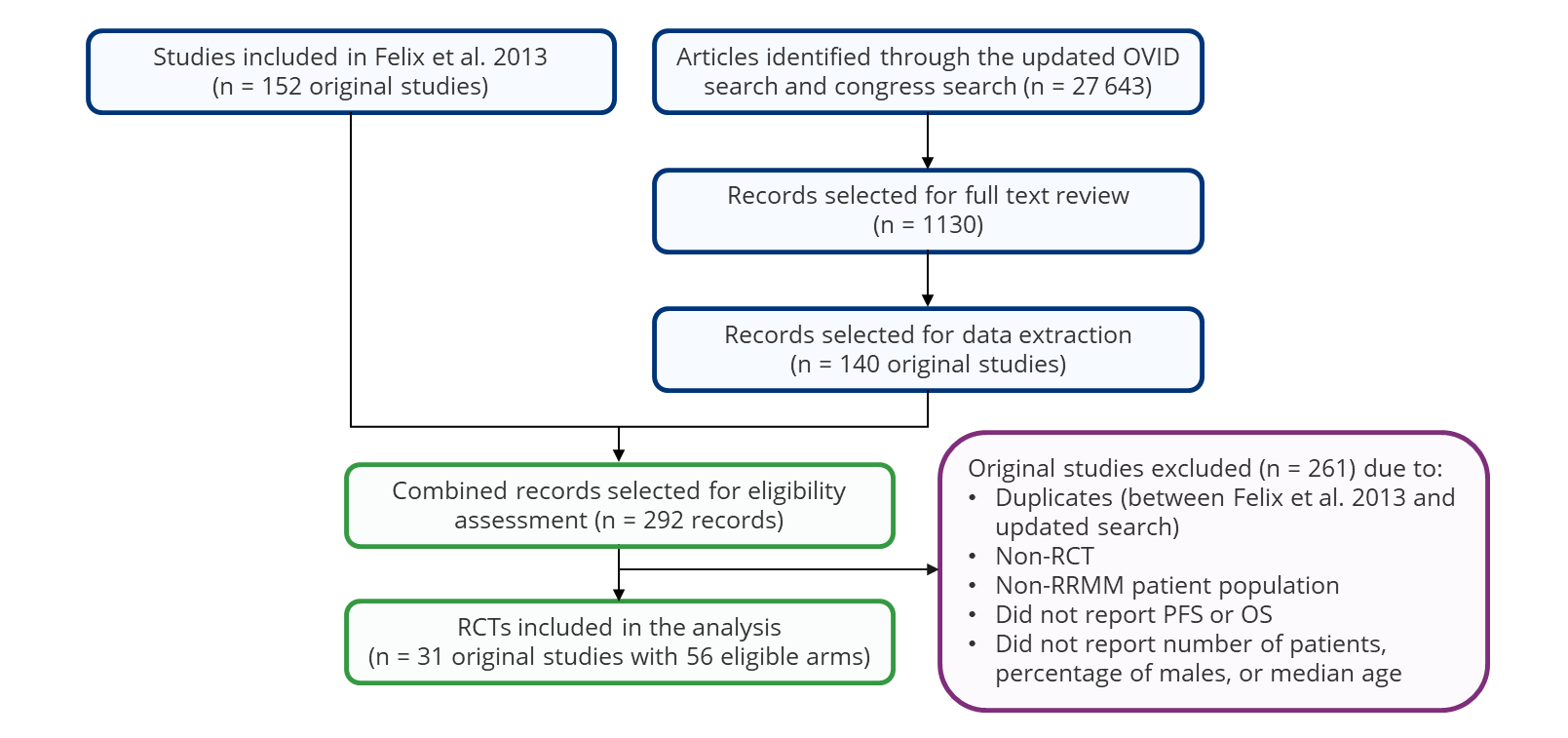
Additional Figure 1: PRISMA flow diagram**

Supplement: Supplementary file 3 — Supplementary Material 3 [file 12885_2024_12263_MOESM3_ESM.docx]
